# Marriage is a dependent risk factor for mortality of colon adenocarcinoma without a time-varying effect

**DOI:** 10.18632/oncotarget.15378

**Published:** 2017-02-16

**Authors:** Minling Liu, Lixian Li, Wei Yu, Jie Chen, Weibin Xiong, Shuang Chen, Li Yu

**Affiliations:** ^1^ Department of Pathology, Zhujiang Hospital, Southern Medical University, Guangzhou, China; ^2^ Department of Biostatistics, School of Public Health, Southern Medical University, Guangzhou, China; ^3^ Department of Oncology, Zhujiang Hospital, Southern Medical University, Guangzhou, China

**Keywords:** marriage, colon adenocarcinoma, time-varying effect, all-cause mortality, surveilance

## Abstract

**Background:**

It has been well recognized that the effects of many prognostic factors could change during long-term follow-up. Although marriage has been proven to be a significant prognostic factor for the survival of colon cancer, whether the effect of marriage is constant with time remain unknown. This study analyzed the impact of marital status on the mortality of colon cancer patients with an extended Cox model that allowed for time-varying effects.

**Methods:**

We identified 71,955 patients who underwent colectomy between 2004 and 2009 to treat colon adenocarcinoma from the Surveilance, Epidemiology and End Results Database. The multivariate extended Cox model was used to evaluate the effect of marital status on all-cause mortality, while the Fine-Gray competing risks model was used for colon cancer-specific mortality, with death from other causes as the competing risk.

**Results:**

The unmarried patients carried a 1.37-fold increased risk of all-cause mortality compared with the married patients (95%CI: 1.33-1.40; p<0.001), and the hazard ratio remained constant over time. Being unmarried was at a higher risk of death from colon adenocarcinoma as well as death from other causes. Four variables including tumor site, tumor grade, sex and TNM stage were proved to have time-varying effects on survival.

**Conclusions:**

Marriage is a dependent prognosis factor for survival of surgically treated colon adenocarcinoma patients. Psychological interventions are suggested to improve receipt of treatment among unmarried patients, as their poor survival may be due to the inefficient treatment.

## INTRODUCTION

Colorectal cancer is the third most common cancer in men worldwide(746,000 cases, 10.0% of the total) and the second most common cancer in women (614,000 cases, 9.2% of the total) [1]. In addition, colorectal cancer is the fourth leading cause of cancer-related deaths, with 693,000 deaths worldwide per year [1]. The survival of this type of cancer is affected by many factors, such as age, grade, stage, tumor site [2], molecular pathogenesis, treatment regimen and socioeconomic status. Marriage, as an important psychosocial factor, has been proven to be a significant prognostic factor for many cancers [3–14]. Previous studies using the Cox proportional hazards regression model (Cox PH model) have demonstrated that being married at the time of diagnosis is associated with a better survival of colorectal cancer [6, 11–14]. These conclusions could be misleading if marital status has a time-varying effect, because the Cox PH model relies on a fundamental proportional hazard (PH) assumption, which is that the relative risks of the covariates do not change over time. However, whether marriage has a time-varying effect on the survival of colorectal cancer patients remains unknown. To investigate this question, we performed this study based on data from the Surveilance, Epidemiology and End Results [15] (SEER) database, with an extended Cox model that allowed for time-varying effects. We tried to make the cohort more comparable by limiting patients who underwent colectomy for colon adenocarcinoma.

## RESULTS

### Patient characteristics

Of the 71,955 patients included in our analyses, 41,126 patients (57.16%) were married and 30,829 (42.84%) were unmarried. There were 16,298(39.63%) deaths in married group, including 11,005 died of colon cancer and 5,293 died due to other reasons. There were 16,232(52.65%) deaths in unmarried group, including 9,749 died for colon cancer and 6,483 died due to other reasons. The clinical and demographic characteristics of the study cohort according to marital status are summarized in Table 1. In general, unmarried patients were, on average, 4.85 years older than married patients and were significantly more likely to be both female and black, have right-side cancer, have higher grade tumors, be at an advanced tumor stage, but be at an earlier AJCC stage (p<0.001). The proportion of patients who presented with metastatic disease was similar between the married and unmarried group (15.0% VS 14.8%, p=0.776), which is inconsistent with previous studies [12, 13]. There was no significant difference in lymph node metastasis between two groups.

**Table 1:** Baseline Characteristics of the study cohort according to Marital Status (N = 71,955)

Characteristic	Married(n=41,126)	Unmarried(n=30,829)	P
Vital status			<0.001
alive	24,828(60.37%)	14,597(47.35%)	
dead for colon cancer	11,005(26.76%)	9,749(31.62%)	
dead for other causes	5,293(12.87%)	6,483(21.03%)	
Age	65.64±12.8	70.49±14.5	<0.001
Race			<0.001
white	33,542(81.56%)	23,805(77.22%)	
Black	3,625(8.81%)	4,966(16.11%)	
Other	3,959(9.63%)	2,058(6.68%)	
Sex			<0.001
Male	23,982(58.31%)	10,485(34.01%)	
female	17,144(41.69%)	20,344(65.99%)	
Tumor site			<0.001
left	17,034(41.42%)	11,281(36.59%)	
right	23,516(57.18%)	19,004(61.64%)	
large intestine, NOS	576(1.40%)	544(1.76%)	
Grade			<0.001
grade I	3,911(9.51%)	2,879(9.34%)	
grade II	29,190(70.98%)	21,436(69.53%)	
grade III	7,441(18.09%)	6,058(19.65%)	
grade IV	584(1.42%)	456(1.48%)	
Stage			<0.001
stage 0/I	9,870(24.00%)	6,432(20.86%)	
stage II	12,357(30.05%)	10,353(33.58%)	
stage III	12,729(30.95%)	9,470(30.72%)	
stage IV	6,170(15.00%)	4,574(14.84%)	
Tumor stage			<0.001
Tis/T0/Tx	570(1.39%)	363(1.18%)	
T1	5,070(12.33%)	2,947(9.56%)	
T2	6,269(15.24%)	4,364(14.16%)	
T3	24,103(58.61%)	18,774(60.90%)	
T4	5,114(12.43%)	4,381(14.21%)	
Nodal stage			0.102
N0	23,200(56.41%)	17,637(57.21%)	
N1	10,247(24.92%)	7,611(24.69%)	
N2	7,672(18.65%)	5,573(18.08%)	
Nx	7(0.02%)	8(0.03%)	
Metastatic disease			0.776
M0	34,953(85.00%)	26,252(85.15%)	
M1	6,170(15.0%)	4,574(14.84%)	
Mx	3(0.01%)	3(0.01%)	
Lymph node rates			0.096
0.00≤LNR<0.17	30,521(74.21%)	22,990(74.57%)	
0.17≤LNR<0.41	5,668(13.78%)	4,301(13.95%)	
0.41≤LNR<0.69	2,839(6.90%)	1,987(6.44%)	
0.69≤LNR	2,098(5.10%)	1,551(5.03%)	

Abbreviations: NOS, not otherwise specified; LNR, lymph node rates.

### Impact of marital status on all-cause mortality

**Figure 1:**
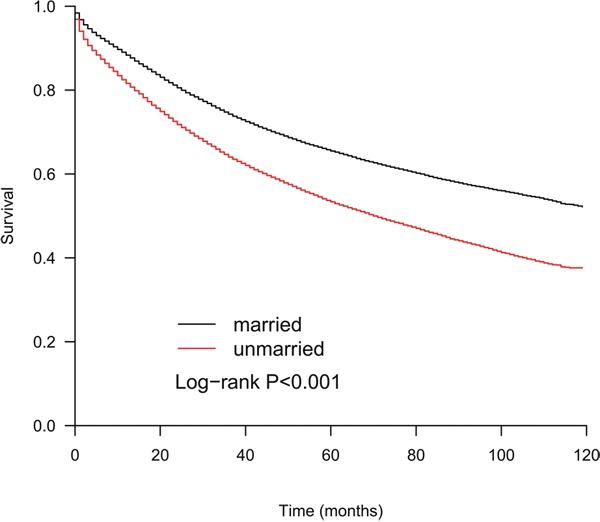
The Kaplan-Meier curves show that the OS of the married patients is better than that of the unmarried patients (p<0.001)

**Figure 2:**
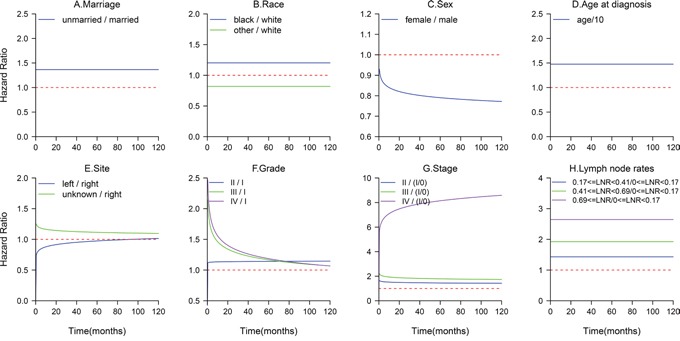
Time-varying effect of each factor on all-cause mortality **A**. The HR of unmarried patients was relatively stable over time. **D, B**. The HR curve of age and race did not exhibit a clearly obvious change with time. **C**. Using females as a reference, the risk associated with the male gender increased over time. **E**. The impact of tumor site decreased over time. **F**. The HR of grade III or IV patients compared with that of grade I patients decreased rapidly within 2 years. **G**. The HR of stage IV patients compared with that of stage I patients increased with a longer survival time. **H**. The effects of LNR on all-cause mortality remained constant.

The median OS of all the patients was 99 months, the median OS of the unmarried group was 70 months and that of the married group was not determined until the last follow-up time(log-rank p<0.001, Figure 1). The findings for all-cause mortality are presented in Table 2. In the univariate analysis, patients who were married at the date of diagnosis survived significantly longer than those who were unmarried (HR: 1.50; 95% CI, 1.47-1.54; p<0.001). Other variables were also strongly correlated with all-cause mortality (all p<0.05, Table 2). Performing a multivariate analysis, both the extended Cox model and Cox PH model showed that increasing age, being black, being male, being unmarried, having a right-side tumor, having a higher tumor grade, having a more advanced TNM stage and a higher LNR were significant adverse prognostic factors(p<0.05, Table 2). In multivariate analysis with the extended Cox model, four variables including tumor site, tumor grade, sex and TNM stage showed time-varying effects on survival, while marriage did not. An unmarried status carried a 1.37-fold increase in the risk of death compared with a married status (95%CI, 1.33-1.40; p<0.001). Figure 2 displays the HR curve of each variable for all-cause mortality over time after adjustment for other variables. The HR curves of marriage, race, age and LNR for all-cause mortality remained relatively stable over time. The impact of tumor site and grade on all-cause mortality decreased over time, while that of sex and TNM stage increased over time.

**Table 2:** Univariate and Multivariate analysis for all-cause mortality

Variable	Cox Proportional Hazard Model						Extended Cox Model			
	Univariate analysis			Multivariate Analysis			Multivariate Analysis			
	HR(95%CI)	p^a^	PH test*	HR(95%CI)	p^b^	PHtest*	BaselineHR(95%CI)	P	Time-VaryingHR(95%CI)	P
age	1.37(1.36-1.39)	<0.001	0.166	1.49(1.47-1.50)	<0.001	0.613	1.48(1.46-1.49)	<0.001		
race										
white	ref						Ref			
black	1.12(1.08-1.15)	<0.001	0.213	1.19(1.15-1.23)	<0.001	0.802	1.20(1.16-1.24)	<0.001		
other	0.76(0.73-0.79)	<0.001	0.074	0.81(0.77-0.85)	<0.001	0.893	0.82(0.78-0.86)	<0.001		
sex										
male	ref						Ref			
female	0.97(0.95-0.99)	0.003	0.028	0.81(0.80-0.83)	<0.001	0.014	0.91(0.85-0.97)	0.002	0.97(0.95-0.99)	0.001
marriage										
married	ref						ref			
unmarried	1.50(1.47-1.54)	<0.001	0.005	1.38(1.34-1.41)	<0.001	0.245	1.37(1.33-1.40)	<0.001		
site										
right	ref						ref			
left	0.84(0.82-0.86)	<0.001	<0.001	0.93(0.91-0.95)	<0.001	<0.001	0.77(0.72-0.82)	<0.001	1.06(1.04-1.08)	<0.001
unknown	1.25(1.15-1.35)	<0.001	0.003	1.18(1.09-1.28)	<0.001	0.067	1.23(1.00-1.51)	0.049	0.98(0.91-1.04)	0.487
grade										
I	ref						ref			
II	1.42(1.36-1.49)	<0.001	0.198	1.11(1.06-1.16)	<0.001	0.136	1.12(0.98-1.29)	0.098	1.00(0.96-1.05)	0.857
III	2.23(2.13-2.34)	<0.001	<0.001	1.32(1.26-1.39)	<0.001	<0.001	1.96(1.70-2.27)	<0.001	0.88(0.84-0.92)	<0.001
IV	2.38(2.17-2.60)	<0.001	<0.001	1.43(1.31-1.57)	<0.001	0.001	2.22(1.76-2.79)	<0.001	0.86(0.80-0.93)	<0.001
stage										
I/0	ref						ref			
II	1.62(1.56-1.68)	<0.001	<0.001	1.49(1.44-1.55) (1.44-1.55)	<0.001	<0.001	1.61(1.42-1.83)	<0.001	0.97(0.94-1.01)	0.169
III	2.32(2.24-2.41)	<0.001	<0.001	1.85(1.78-1.93) (1.78-1.93)	<0.001	<0.001	2.14(1.89-2.42)	<0.001	0.96(0.92-0.99)	0.015
IV	8.29 (7.99-8.61)	<0.001	<0.001	6.86(6.57-7.16)	<0.001	0.031	5.94(5.26-6.71)	<0.001	1.08(1.04-1.12)	<0.001
LNR										
0.00≤LNR<0.17	ref						ref			
0.17≤LNR<0.41	2.11(2.05-2.17)	<0.001	<0.001	1.41(1.36-1.46)	<0.001	0.168	1.43(1.38-1.48)	<0.001		
0.41≤LNR<0.69	3.12(3.01-3.23)	<0.001	<0.001	1.90(1.82-1.97)	<0.001	0.798	1.92(1.85-2.01)	<0.001		
0.69≤LNR	5.23(5.04-5.43)	<0.001	<0.001	2.59(2.48-2.71)	<0.001	0.046	2.64(2.53-2.77)			

Abbreviations: HR, hazard ratio; CI, confidence interval; LNR, lymph node rates.

* Grambsch - Therneau proportional hazards test

a: In univariate analysis, all variables were strongly correlated with all-cause mortality (all p<0.05)

b:we conducted non-proportionality test in univariate analysis with Cox PH model, and we identify that sex, marriage, tumor site, tumor grade, tumor stage, lymph node rates were time-dependent factors (p<0.05). But when we conducted non-proportionality test in multivariate analysis with Cox PH model, we identify that marriage, lymph node rates satisfied the PH assumption (p>0.05).

c:A multivariate analysis with a time-dependent Cox model for all-cause mortality was performed using the following factors: age, race, sex (*log t), marriage, tumor site(*log t), tumor grade(*log t), tumor stage(*log t), lymph node rates.

### Impact of marital status on cancer-specific mortality

**Figure 3:**
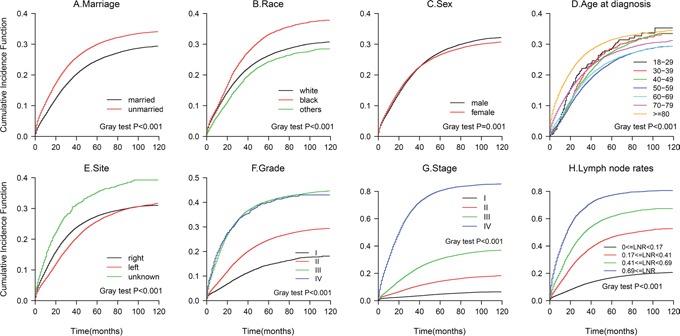
Cumulative Incidence Function (CIF) of deaths from colon adenocarcinoma

**Figure 4:**
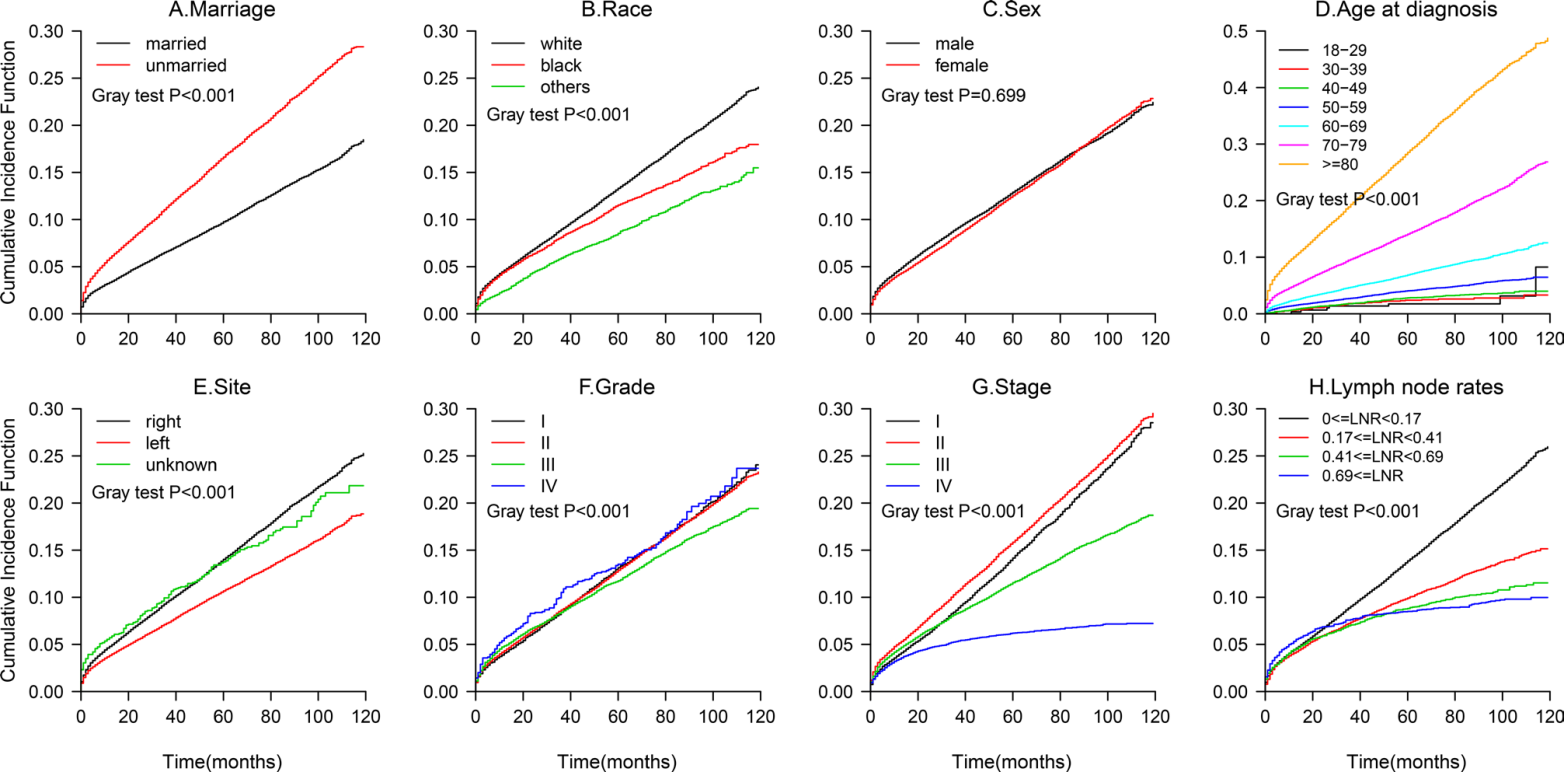
Cumulative Incidence Function (CIF) of deaths from other causes

The results from the multivariate Fine and Gray competing risks regression model are shown in Table 3. The CIF curves are plotted in Figure 3. The 5-year cancer-specific mortality rate was 24.76% for the married patients and 30.01% for the unmarried patients (p<0.001). Marital status was a significant independent predictor of colon cancer death, with an 20.7% increased risk of cancer-specific mortality for the unmarried patients compared with that for the married patients(HR,1.21; 95%CI, 1.17-1.24; p<0.001). The unmarried patients also had a greater probability of death from other causes (data not shown, Figure 4). An older age, being black, being male, having a right-side tumor, and a higher grade or TNM stage were associated with an increased risk of death from colon cancer (all p<0.001), findings that were similar to the results of the all-cause mortality analysis.

**Table 3:** Fine and Gray Proportional Hazards Regression Analysis of colon cancer-specific mortality

Variable/ Characteristic	HR(95%CI)	P
age	1.18(1.17-1.20)	<0.001
race		
white	ref	
black	1.21(1.16-1.26)	<0.001
other	0.88(0.83-0.93)	<0.001
sex		
male	ref	
female	0.92(0.90-0.95)	<0.001
marriage		
married	ref	
unmarried	1.21(1.17-1.24)	<0.001
site		
right	ref	
left	0.92(0.90-0.95)	<0.001
unknown	1.14(1.02-1.27)	0.016
grade		
I	ref	
II	1.14(1.07-1.21)	<0.001
III	1.44(1.35-1.54)	<0.001
IV	1.41(1.25-1.60)	<0.001
stage		
I/0	ref	
II	2.86(2.65-3.07)	<0.001
III	5.20(4.83-5.59)	<0.001
IV	21.42(19.90-23.06)	<0.001
LNR		
0.00≤LNR<0.17	ref	
0.17≤LNR<0.41	1.47(1.41-1.52)	<0.001
0.41≤LNR<0.69	1.93(1.85-2.02)	<0.001
0.69≤LNR	2.47(2.34-2.60)	<0.001

Abbreviations: HR, hazard ratio; CI, confidence interval; LNR, lymph node rates.

Hazard ratios (95% CIs) were derived from the competing risks model that was controlled for all variables mentioned in the above.

## DISCUSSION

Using the SEER database, we identified 71,955 patients who underwent colectomy for colon adenocarcinoma. Kaplan-Meier curves showed that the median OS of the unmarried group was significantly shorter than that of the married group (70months vs more than 120months). When we performed a multivariate analysis with an extended Cox model, we found that the unmarried patients carried a 1.37-fold increased risk of all-cause deaths compared with the married patients, and this risk remained constant over time. Further analysis showed that being unmarried was at a higher risk of death from colon adenocarcinoma as well as death from other causes.

Why the married status result in more favorable OS is not totally clear yet. A recent research has observed that the survival benefits of marriage may not because of better material resources, including health insurance status and neighborhood socioeconomic status [16]. The lower colorectal cancer screening rate [17] [18, 19], the higher metastatic cancer rate [12] [13]and the lower surgery rate [13]in unmarried group may be some potential reasons, because the early detection and early treatment can reduce mortality of cancer. But why the marriage continues to play a role even after surgery? In our opinion, it may be due to the inefficient treatment. For patients with surgically treated stage III colon cancer, the initiation and completion of adjuvant chemotherapy was largely influenced by marital status [20], and the chemotherapy use was lower in divorced and widowed patients [21]. Besides, marital status have also been proven a significant variable associated with advancing through second- and subsequent-line treatments among metastatic colon cancer patients [22]. This issue needs to be looked at in future studies.

We recommend psychological supports for unmarried patients, as high levels of distress were found among unmarried colorectal cancer patients [23]. Although recent researches have failed to detect a survival benefit among patients who received psychological intervention [24–28], most of them did not further analyze unmarried patients. Those results could not be transferred straight forward. Whether unmarried patients will benefit from psychosocial intervention should be further explored.

It has been well recognized that prognostic effects could change during long-term follow-up. These prognostic factors include age [29–31], tumor stage [31], tumor grade [32–34], tumor size [30, 33, 35, 36], nodal status [30, 33, 36], hormone receptor status [32, 34, 35, 37], gene mutations [38], tumor marker status [39], drug exposure and chemotherapy [30, 40, 41]. In this study, four variables including tumor site, tumor grade, sex and TNM stage were also proved to have time-varying effect on all cause mortality. The Cox PH model may lead to biased estimates for time-varying factors since it assume that the relative risks of the covariates do not change over time. Quantin C et al had compared Cox PH model and some non-proportional hazard survival models in modeling the impact of prognostic factors on all-cause mortality in colon cancer, found that the effects of most clinical prognostic factors are non-proportional, illustrated that non-proportional survival models are more appropriate and better understanding the time-dependent aspect of prognostic factors [31].[42].

Due to the nature of these data, potential limitations of our study should be considered. First, we could not examine information about chemotherapy or other complementary treatments which could affect mortality. Second, the molecular markers of prognosis, such as microsatellite instability, immune cell infiltration, RAS and other gene mutations, were not presented in the database [43]. Third, changes in the marital status that occurred since the diagnosis are not available in this database. We were unable to account for those markers in our multivariate model.

Despite these potential limitations, we have found that unmarried patients were associated with a higher all-cause mortality risk, and this HR did not change over time. Being unmarried was at a higher risk of death from colon adenocarcinoma as well as death from other causes. Clinicians should assess marital status as a marker of prognosis of colon cancer. Our study points to the need for psychological interventions to improve receipt of treatment among unmarried patients.

## MATERIALS AND METHODS

### Data source

We collected data from the November 2015 submission of the SEER Database (http://www.seer.cancer.gov/) using the SEER*Stat software (Version 8.3.2). Sponsored by the National Cancer Institute, the SEER program includes cancer cases diagnosed from 1973 to 2013, collects data on patient demographics, tumor characteristics, limited treatment information, and survival information. It includes patients from 18 SEER registries, covers approximately 30 percent of the US population, and is an authoritative source of information on cancer incidence and survival in the US.

First, the initial cohort of 78,647 patients who underwent colectomy between 2004 and 2009 to treat colon adenocarcinoma was identified according to the following filter conditions. The patients with colon adenocarcinoma were identified using the International Classification of Diseases for Oncology, 3rd Edition (ICD-O-3/WHO 2008) site codes as “colon excluding rectum” and the ICD-O-3 morphology codes 8140-8141, 8143, 8145,8147, 8210-8211, 8220-8221, 8255, 8260-8263, 8310, 8323, 8480-8481, and 8570-8576, while the patients who underwent colectomy were enrolled using site-specific surgery codes 30, 40, 50, 60, 70, and 80. The period was restricted from 2004 to 2009, during which cancers were classified based on the American Joint Committee on Cancer (AJCC) 6th edition staging criteria. We excluded patients with an unknown marital status, those with two or more malignant tumors, those who were alive or had died without a survival time, and those diagnosed by death certificate or autopsy only.

Of 78,647 patients, we further excluded patients who were less than 18 years of age (N=14), those lacking a histological grade (N=3,528), those whose stage was coded as “unknown” or “not applicable” (N=849), those without lymph a node examination, those cases in which the number of nodes examined or whether the nodes were examined was not known (N=2,042), those with an unknown number of positive lymph nodes or nodes that were not known to be positive or negative(N=33), and those with an unknown race or ethnicity (N=226), leaving 71,955 patients for the survival study.

### Variables of interest

We obtained information routinely recorded at diagnosis for each patient, including age, sex (male vs female), marital status (married vs unmarried), race (white, black, other), tumor site, histological grade, T classification, N classification, M classification, and TNM stage (AJCC 6th), the total number of lymph nodes removed, the total number of positive lymph nodes, vital status, the underlying cause of death from the death certificate, and survival months. When we limited the cancer site to the colon, excluding the rectum, the following 9 sites were included in the SEER database: the appendix, cecum, ascending colon, hepatic flexure, transverse colon, splenic flexure, descending colon, sigmoid colon, and large intestine, (not otherwise specified). The tumor sites were divided into three groups, left-side tumors (splenic flexure to sigmoid descending colon), right-side tumors (appendix to transverse colon), and large intestine, NOS. Patients who were single, separated, divorced, or widowed at diagnosis were divided into the unmarried group, while patients who were married (including common law marriages) were divided into the married group. In the current study, we calculated a new variable, lymph node ratio (LNR), which was defined as the number of positive lymph nodes divided by the number of lymph nodes examined. LNR has been proven to be a strong independent prognostic factor for colon cancer [44–46], and the cut-off values of 0.17, 0.41 and 0.69 were recommended for the risk group stratification [44–46].

### Outcome of interest

The follow-up time was calculated from the date of diagnosis to death or the date the study ended (December 31, 2013). Deaths from any cause were used as primary events, while deaths from colon adenocarcinoma were considered as secondary events. We calculated the overall survival (OS) as the number of months from the date of diagnosis until death from any cause. All the patients who remained alive on December 31, 2013 were considered censored.

### Statistical analysis

The baseline characteristics of the study cohort according to survival status were compared using a t-test for continuous variables and a chi-squared test for categorical variables. Survival curves were constructed using the Kaplan-Meier method and were compared using the log-rank test for each factor in a univariate analysis. All factors were verified for the assumption of the Cox PH model using Therneau-Grambsch PH tests. A p-value<0.05 indicated a violation of the PH assumption. As shown in Table 2, in the events of OS, some variables did not meet the assumption (p<0.05). Therefore, we further multivariate analysis on all-cause mortality using an extended Cox model (a simple extension with a time-varying coefficient model) [47]. T classification, N classification, and M classification were not included in the multivariate analysis due to the collinearities with TNM stage. The extended Cox model was adjusted for age, race, sex, marriage, tumor site, tumor grade, tumor stage, lymph node rates. For variables with time-varying effects, their hazard ratio (HR) at a given time(t) was calculated using the following formula: HR(t)=HR_constant-effect_×(HR_time-varying-effect_^log(t)). The Fine-Gray competing risks model was utilized to determine cancer-specific mortality, with death from other causes as the competing risk. The probabilities of cancer-specific mortality and competing risk mortality were described using the cumulative incidence function (CIF), which was compared using Gray's test between the groups. Statistical analyses were performed using the R software (version 4.3.2). All the statistical tests were 2-tailed and were performed at a p-value less than 0.05.
